# Peripheral nerve stimulation for the treatment of chronic knee pain

**DOI:** 10.1038/s41598-023-42608-x

**Published:** 2023-09-20

**Authors:** Anton Früh, Tarik Alp Sargut, Abdelhalim Hussein, Bartolomäus Muskala, Anja Kuckuck, Melanie Brüßeler, Peter Vajkoczy, Simon Bayerl

**Affiliations:** 1 Department of Neurosurgery, Charité - Universitätsmedizin Berlin, Corporate Member of Freie Universität Berlin, and Humboldt-Universität Zu Berlin, Berlin Institute of Health, Berlin, Germany; 2 Berlin Institute of Health, BIH Academy, Junior Digital Scientist Program, Charité-Universitätsmedizin Berlin, Berlin, Germany; 3 Helios Albert-Schweitzer-Klinik, Northeim, Germany; 4 Inter Neuro, Private Practice, Berlin, Germany; 5 Ludwig-Maximilians-University Munich, Munich, Germany

**Keywords:** Chronic pain, Peripheral neuropathies

## Abstract

The average age of our population is increasing, resulting in a high incidence of chronic degenerative knee pathologies. Several treatment options, including surgical procedures are available to help mitigate these pathologies. However, the percentage of subjects with chronic post-surgical knee pain is still estimated at 16–20%. Neuromodulation techniques such as spinal cord stimulation and dorsal root ganglion stimulation (DRGS) are treatment options for subjects with chronic knee pain. The evidence for peripheral nerve stimulation (PNS) is minimal due to a limited number of neuromodulation systems capable of targeting the distal part of the lower limbs. This study aimed to investigate the safety and efficacy externally powered PNS systems for the treatment of chronic intractable knee pain targeting the saphenous nerve. Patients suffering from chronic intractable post-surgical knee pain received landmark-guided peripheral nerve stimulation of the branches of the saphenous nerve. All implants were performed with an externally powered PNS system to avoid lead migration as a result of cross-joint lead positions tunneling towards an Implantable Pulse Generator to the trunk. Data were collected retrospectively. Subject-reported outcome was measured via numerical rating scale values on a 10-point scale measuring pain intensity at rest and in motion. Additional data were collected for the subjects treated at the Charité location, including quality of life with the SF-36 form, quality of sleep with the Pittsburgh Sleep Quality Index and mood states with the short form of the General Depression Scale. Thirty-three patients received direct to permanent implant, landmark-guided peripheral nerve stimulation of the saphenous nerve branches. Six (18.2%) subjects reported non-sufficient initial benefit from the therapy and were explanted. Two subjects were explanted due to wound infections. The total study population reported included 25 patients. These subjects reported significant improvements related to pain, quality of life, mood quality, and quality of sleep. Additionally, subjects were able to reduce their opioid medication significantly after PNS therapy. Externally powered PNS at the saphenous nerve branches is a straightforward, selective and safe technique for patients with chronic knee pain. The landmark-guided implantation technique is less invasive than classical neuromodulation techniques such as spinal cord or DRGS and complication rates remain low. Short-term results are promising and show considerable reductions in pain scores and opioid intake. Long-term results are pending.

## Introduction

The average age of our population is increasing, resulting in a high incidence of chronic degenerative knee pathologies. Total knee replacement surgery (TKA) is a frequently performed elective procedure indicated for subjects with knee pain, refractory to conservative therapies^1^. In the United States alone, about 600,000 total knee arthroplasties are performed annually, with a significant increase projected by 2030^2^. TKA is a reliable surgical procedure with positive long-term results^3^. However, 16–20% of all patients report chronic pain after the intervention^4^. Chronic post-surgical knee pain can intensify beyond the initial healing phase and even increase in intensity^5,6^. Affected subjects experience severe functional limitations and decreased quality of life. Limited evidence is available today to effectively treat these subjects^7^. Treatment of post-operative pain that is not structural in nature proves to be a challenge for clinical caregivers^8^. Furthermore revision surgeries have limited success rates when performed solely for pain^9^. Due to the increasing concern for the overuse/abuse of opioid medication, there is a strong need for suitable and effective treatment alternatives^1^.

Neuromodulation techniques like spinal cord stimulation (SCS) and/or dorsal root ganglion stimulation (DRGS) have been successfully used in the treatment of chronic knee pain^1,4,8,10,11^. The evidence involving peripheral nerve stimulation (PNS) is minimal due to the limited number of neuromodulation systems capable of targeting the distal part of lower limbs.

In this study, we seek to investigate the safety and efficacy of an externally powered PNS system in the treatment of chronic intractable knee pain at the saphenous nerve.

## Materials and methods

This clinical, retrospective multicenter trial was approved by the local ethics committee of Charité University Hospital (ethical approval number: EA2/093/13). We retrospectively included subjects suffering from chronic intractable post-surgical knee pain refractory to a multimodal pain management paradigm. Patients received landmark-guided peripheral nerve stimulation of the saphenous nerve branches at Charité Berlin (providing specialty care to a population of about 3.5 million people), Helios Hospital Northeim and a private practice. All methods were carried out in accordance with relevant guidelines and regulations. Informed consent was obtained from all subjects.

### Data collection and outcome measurements

Data were analyzed retrospectively. Outcome scores and pain medication were reported at admission and follow-up visits after the peripheral nerve stimulator system implantation. Subject-reported outcome was measured via numerical rating scale (NRS) values on a 10-point scale measuring pain intensity at rest and in motion. Additional measurements were reported for subjects that were treated at Charité, including quality of life with the SF-36 form^12^, quality of sleep evaluated with the Pittsburgh Sleep Quality Index (PSQI)^13^ and mood states with the short form of the General Depression Scale (ADS-K)^14^.

### Deployed devices

In this study Freedom® systems (Curonix LLC, USA) in a manner analogous to comparable studies were used^15–17^. These PNS devices utilize high-frequency electromagnetic coupling technologies to energize implanted neurostimulators. The stimulators consist of either 4- or 8-contact electrode arrays. Compact and rechargeable transmitters provide both energy and the data to the neurostimulators. The systems employ pulsed electric currents to generate electrical fields to suppress transmission of pain signals. The simulation parameters included a pulse width of 32 μs and a frequency of 1499 Hz. The amplitudes were adjusted and varied based on patients preferences.

### Surgical technique

**Figure 1:**
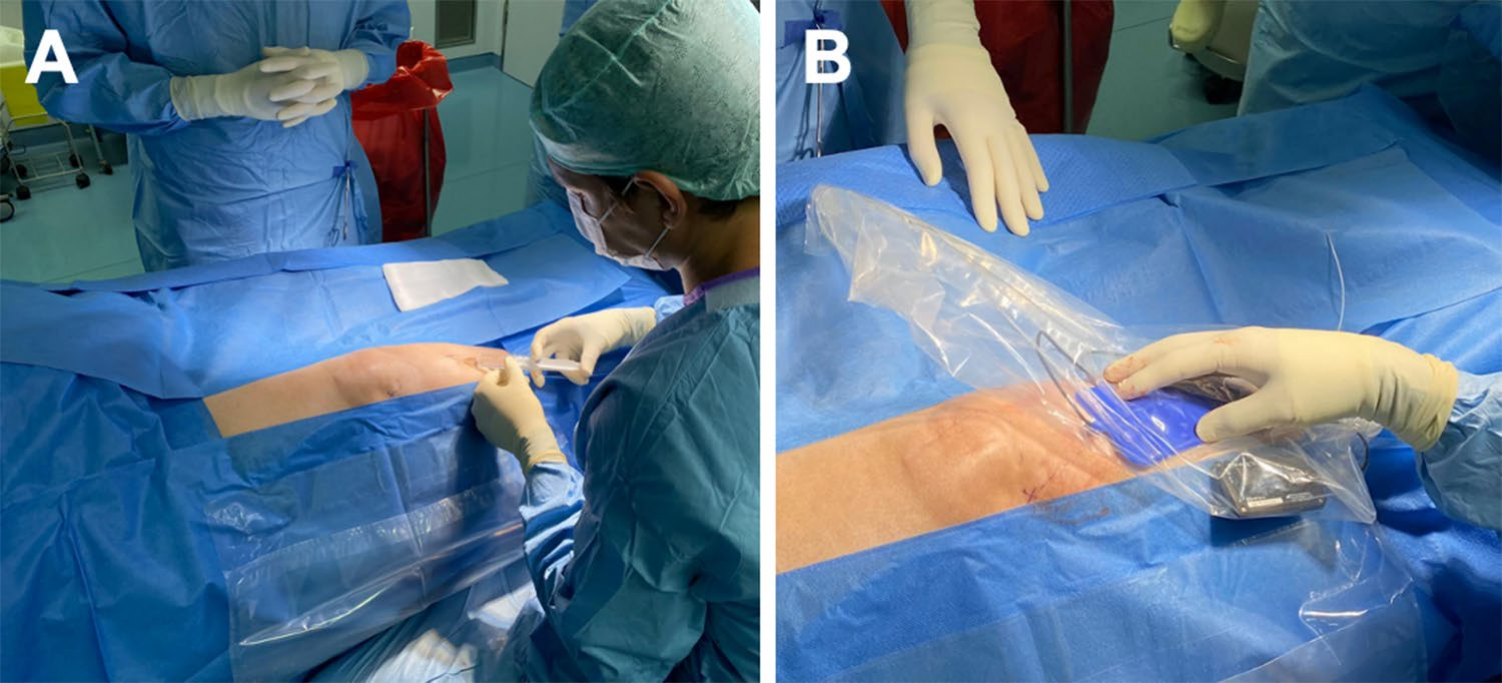
Surgical procedure of an 80-year-old male subject suffering from osteoarthritis. (**A**) Injection of local anesthesia, (**B**) stimulation of the infrapatellar ramus of the saphenous nerve.

All surgeries were performed using local anesthesia (Fig. 1A). Subjects were placed supine with a pillow under the targeted knee, and the Infrapatellar saphenous (IPS) nerve area was identified with fluoroscopic guidance. The 4-contact tined electrode array was placed on the skin for planning purposes with the most distal electrode at the IPS nerve. A marking pen was used to map out the trajectory. The first one cm incision was made about ten cm caudal to the target, and an introducer needle was advanced under the skin until the tip was at the desired location. The introducer stylet was removed, and the electrode array was advanced through the introducer until it reached the tip of the catheter. The steering stylet was removed, and a separate receiver was connected to the electrode array. The introducer was removed, and intraoperative testing confirmed stimulation in the painful area (Fig. 1B).

A second incision was made to create a receiver pocket about ten cm caudal to the first incision, and the stimulator was tunneled beneath the skin to the pocket. A knot was tied to connect the separate receiver and electrode array permanently. The distal portion of the neurostimulator was coiled and sutured to itself. The coil was then anchored to the fascia with multiple sutures in the pocket to prevent migration. The pockets were closed with subcutaneous and subcuticular sutures. Tegaderm (3 M, Maplewood, MN) was placed over the incision.

### Statistical analysis

Statistical analysis was performed with SPSS version 25 (IBM Corp), Microsoft Excel 2021 and GraphPad Prism 8.4.2. Discrete data were presented as raw data and means and were analyzed, using the chi-square test. Continuous data were presented as median and interquartile range (IQR) and compared using Wilcoxon matched-pairs signed rank test. Two-sided *p* values < 0.05 were measured to indicate statistical significance.

## Results

### Study population

**Figure 2:**
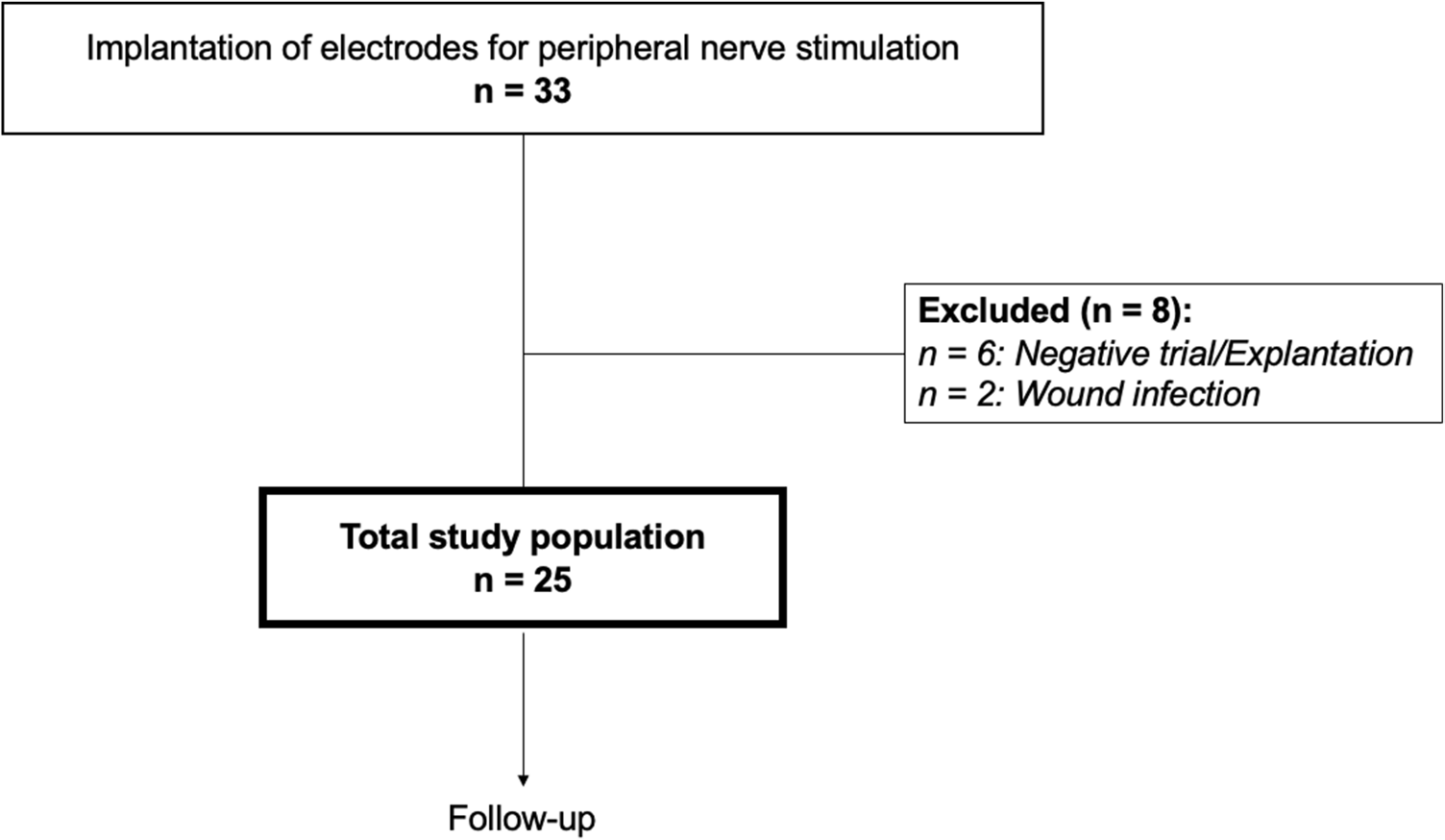
Study flow-chart. *n* number.

Thirty-three subjects suffering from chronic intractable chronic intractable post-surgical knee pain with a median age of 58 (IQR 51–71) years received landmark-guided peripheral nerve stimulation of the saphenous nerve branches. Table 1 provides the baseline characteristics of all patients who had electrodes implanted. Six (18.2%) subjects reported non-sufficient initial benefit (within the first 4 weeks after implantation) and were therefore, explanted. In two subjects (6.1%) stimulators were explanted due to wound infections. The total study population presented included 25 subjects. Figure 2 provides the flowchart of the present study. The median age of the study population was 57 (50–67). Fifty six percent of the subjects were female.

**Table 1:** Baseline characteristics of all patients who had electrodes implanted (n = 33).

	33 patients
Age, years, mean $\pm \hspace{0.17em}$SD (years)	58 $\pm \hspace{0.17em}$15
Female sex, n (%)	15 (45.5)
Pathology, n (%)	
Osteoarthritis stage IV	27 (81.8)
Meniscus/cruciate ligament injury	3 (9.1)
Fracture	2 (6.1)
Nervus saphenous injury after stripping of the vena saphenous	1 (3.0)
BMI, mean $\pm \hspace{0.17em}$SD (kg/m^2^)	29.1 $\pm \hspace{0.17em}$6.5
Preoperative ASA score, n (%)	
ASA score 1	1 (3.0)
ASA score 2	24 (72.7)
ASA score 3	8 (24.2)
Medical history, n (%)	
Diabetes	6 (18.2)
Hypertension	11 (33.3)
Coronary artery disease	7 (21.2)
Chronic heart failure	5 (15.2)
Chronic renal failure	4 (12.1)
COPD	6 (18.2)
History of smoking	10 (30.3)
Permanent intake of ASS 100 mg	8 (24.2)

*ASA* American society of Anesthesiologists, *ASS* acetyl salicylic acid, *COPD* chronic obstructive pulmonary disease, *n* number.

**Figure 3:**
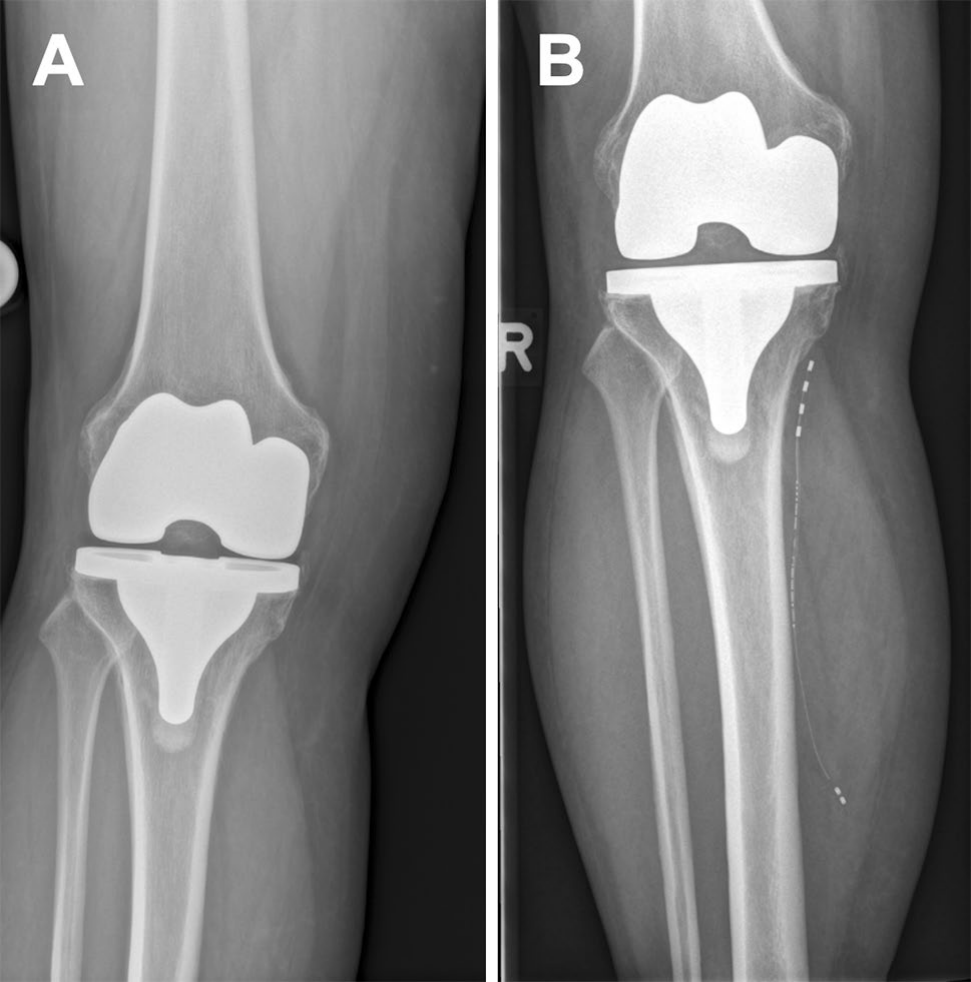
This is a 78-year-old subject suffering from chronic knee pain after total knee arthroplasty due to osteoarthritis. (**A**) Preoperative X-ray. (**B**) Postoperative x-ray with saphenous stimulation system in-situ.

For most patients of the study population, pain was a result of knee arthroplasty for pain due to osteoarthritis (76%), followed by meniscus/cruciate ligament injuries (12%), fractures (8%) and injury of the nervus saphenous after stripping of the vena saphenous (4%). Figure 3 shows exemplary pre- and post-operative x-rays of an included subject with the PNS system in-situ at the Saphenous nerve (Fig. 2).

### Surgical outcome and complications

There were no intra-operative surgical complications to report. Furthermore, none of the subjects reported new post-operative sensomotoric deficits and/or needed additional surgical treatment during the follow-up time. Two subjects suffered from postoperative wound infections.

### Improvement of pain

**Figure 4:**
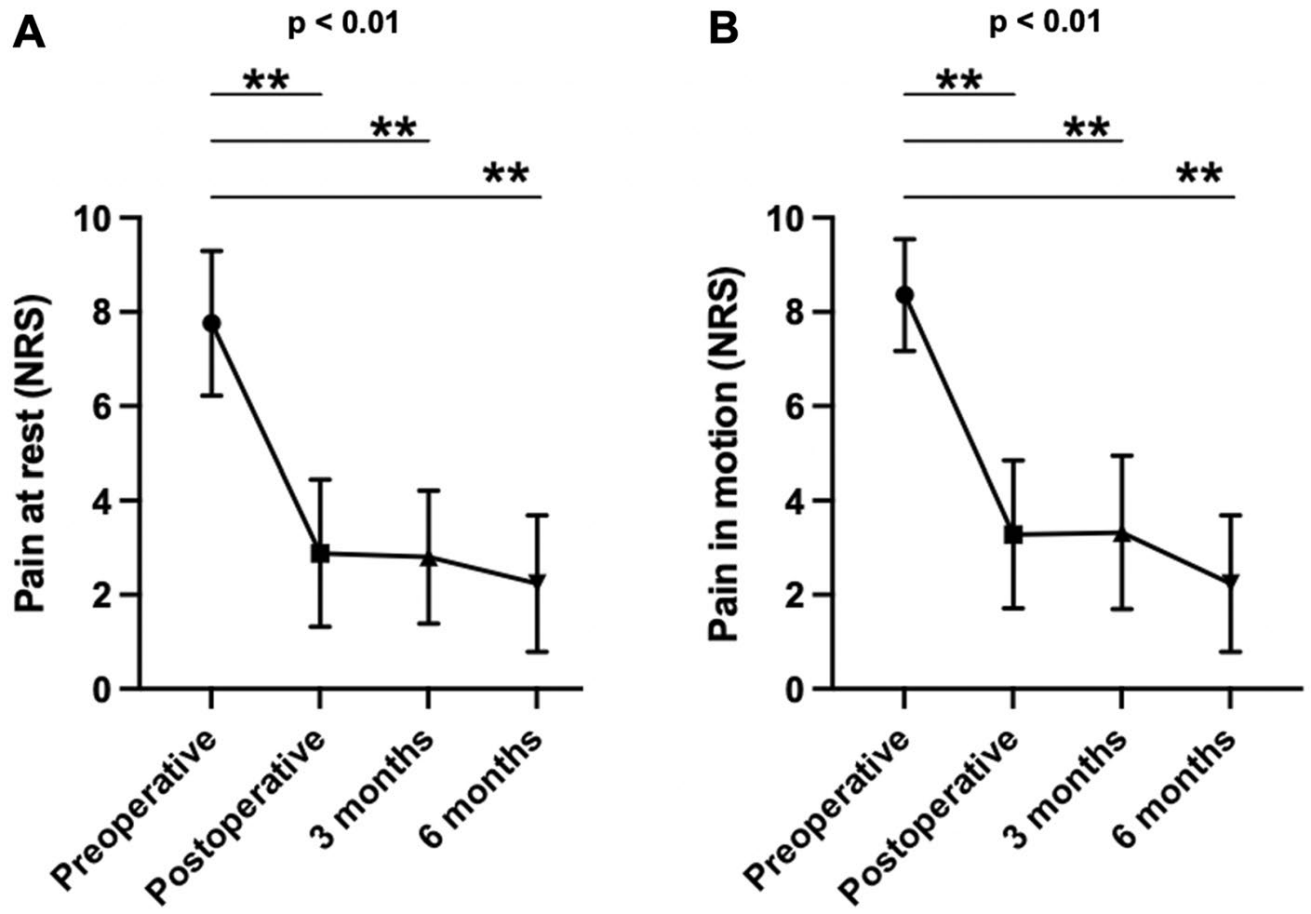
Median knee pain within the first 6 months after surgery (25 patients). (**A**) Pain at rest. (**B**) Pain in motion. *p < 0.05; **p < 0.01 (Wilcoxon matched-pairs signed rank testing).

Initial knee pain showed a significant reduction both during motion and rest. NRS scores decreased from a median of NRS_rest,preop_ = 8 (7–9) to NRS_rest,postop_ = 3 (2–4) (*p* < 0.01). Pain reduction remained consistent at the 3-month [NRS_rest,3 months_ = 2 (2–3), *p* < 0.01] and 6-month follow-ups [NRS_rest,6 months_ = 2 (1–4), *p* < 0.01]]. Figure 4 illustrates the course of knee pain throughout the study.

**Figure 5:**
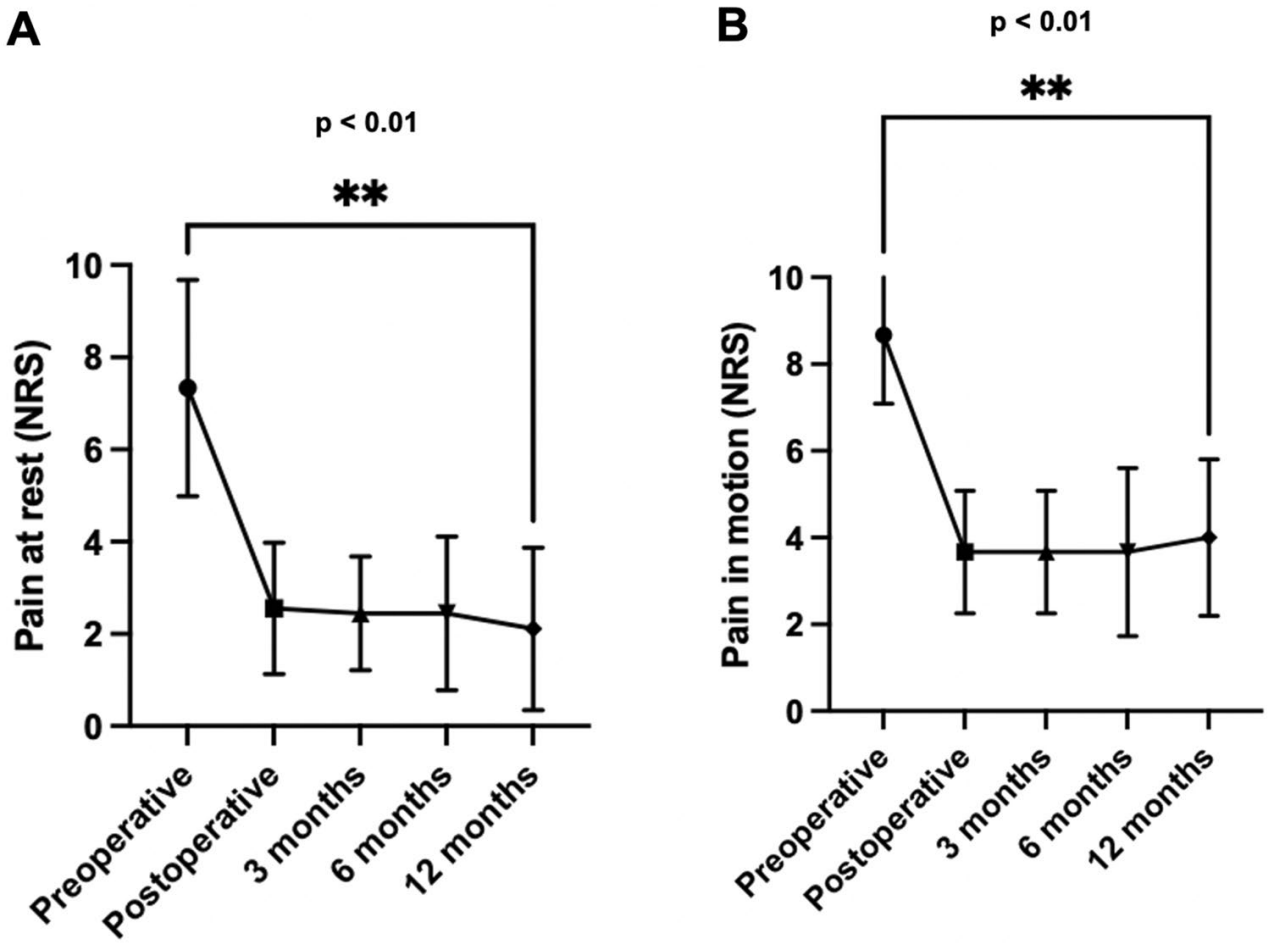
Median knee pain in subjects with long-term follow-up (9 patients). (**A**) Pain at rest. (**B**) Pain in motion. *p < 0.05; **p < 0.01 (Wilcoxon matched-pairs signed rank testing).

Nine subjects underwent an additional 12-month follow-up after the systems’s implantation. They show a persistent significant decrease in knee pain, both at rest (*p* < 0.01) and in motion (*p* < 0.01). Figure 5 demonstrates the knee pain of these patients.

When considering patients who initially did not benefit from the PNS system (“trial phase”) and those who had system explantations due to wound infections, an intention-to-treat analysis revealed an overall success rate with a minimum pain improvement of 50% in 75.8% of all patients.

### Effect on opioid medication

**Figure 6:**
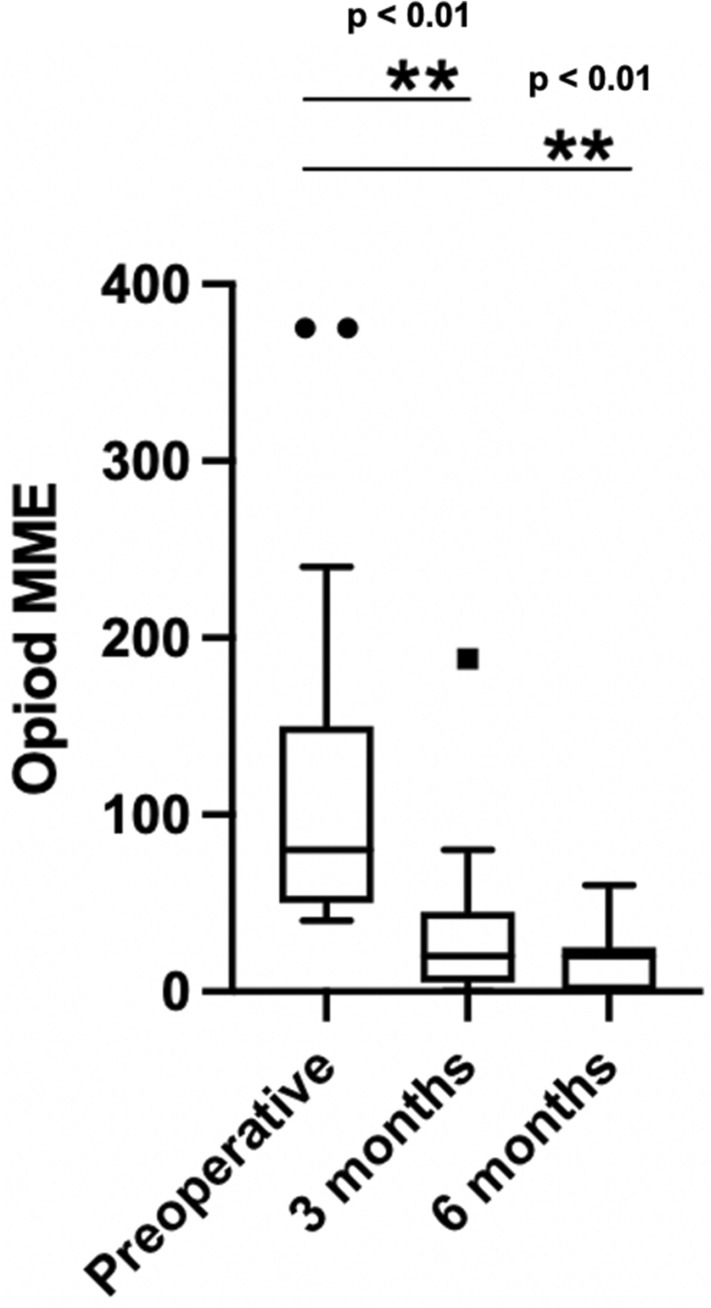
Median Pain medication (MME) intake (25 patients) *p < 0.05; **p < 0.01 (Wilcoxon matched-pairs signed rank testing).

Analysis of pain medication usage showed that subjects treated with PNS were able to reduce their opioid medication from a median of 80 (IQR 50–150) Morphine Milligram Equivalents (MME) preoperatively to 20 (IQR 5–45) MME 3 months and 20 (IQR 0–25) 6 months post-permanent implant. Figure 6 shows the mean opioid medication reduction.

### Improvement of quality of life, sleeping quality and mood

**Figure 7:**
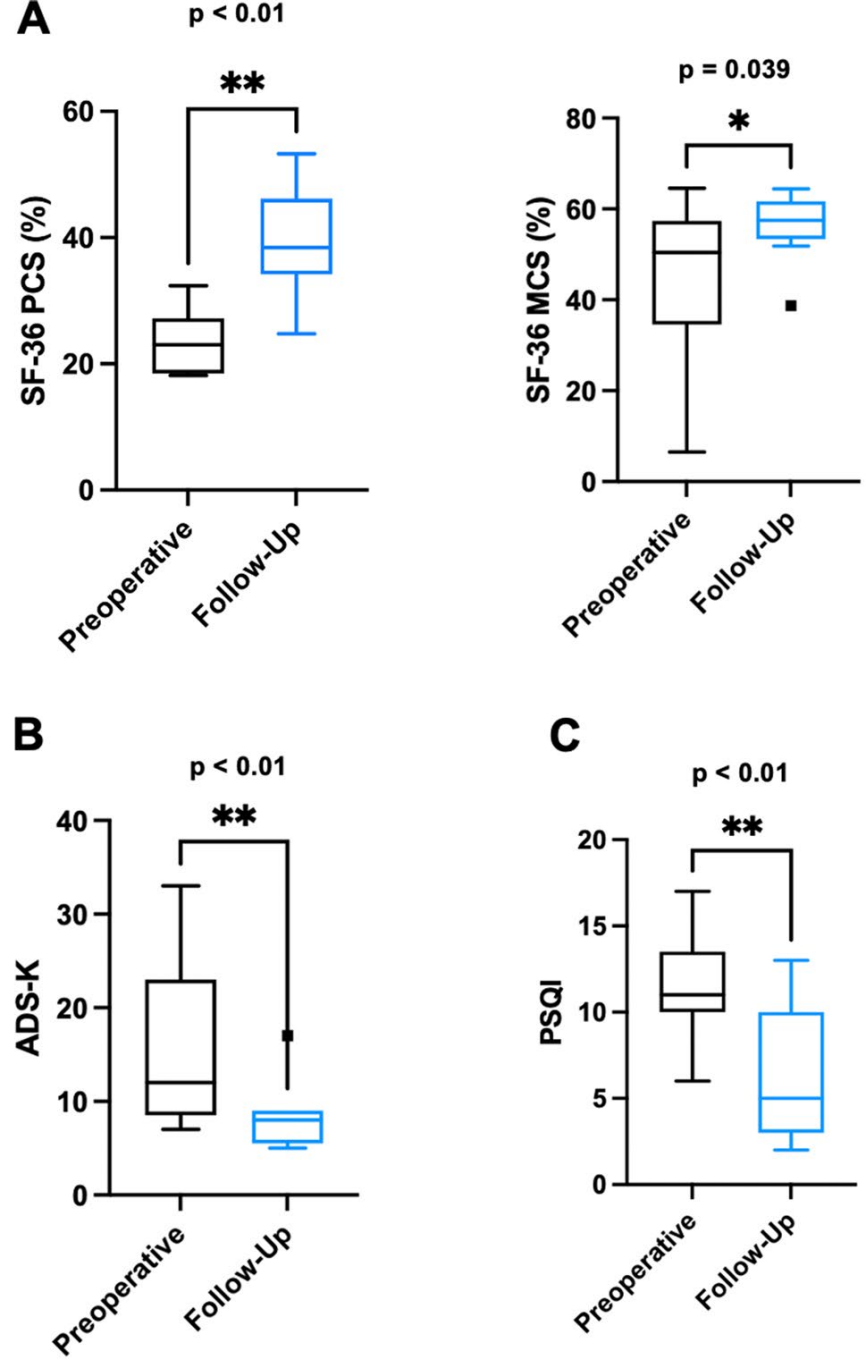
Functional outcome parameters in subjects that were treated at the Charité Center (11 patients). (**A**) Quality of life assessment using SF-36 (0, maximal limitations—100, minimal limitations). (**B**) Mood status using ADS-K questionnaire: ADS-K ≥ 17, depressive disorder, (**C**) Quality of sleep using PSQI questionnaire: 0, no difficulty in sleeping behavior—21, severe difficulties. *p < 0.05; **p < 0.01 (Wilcoxon matched-pairs signed rank testing).

The data shows a significant improvement in quality of life, sleeping quality and mood status of subjects treated at the Charité Centre (n = 11). The median follow-up time for the evaluation was 13 (IQR 12–16) months. Quality-of-life assessment via SF-36 showed a significant improvement in the physical Component Summary Score (PCS) (Median PCS_PreOP_ = 23.05, Median PCS_FollowUp_ = 38.42, *p* < 0.01) and Mentally Health Component Summary Score (MSC) (Median MCS_PreOP_ = 50.42, Median MCS_FollowUp_ = 57.50, *p *= 0.0391). Furthermore, the subjects report a significant improvement as related to the mood state (*p* < 0.01) and quality of sleep (*p* < 0.01). Figure 7 summarizes the functional outcome of the analyzed subjects.

## Discussion

The main finding of this study is that externally powered PNS at the branches of the saphenous nerve appears to be a simple, selective and safe technique for patients suffering with post-surgical chronic knee pain. While there is currently limited evidence for effective treatments for these patients, our data suggest benefits in terms of pain relief, opioid reduction and quality of life.

Persistent knee pain following TKA is a significant complication that is challenging to treat^10^. The current data shows that the subject collective presents a high disease burden, suffering from knee pain with a median NRS of 8 (7–9) and a median intake of 80 MEE. Pain burden is in line with other retrospective studies investigating neurostimulation approaches for knee-pain therapy^11,18^. The present data shows that subjects with neuropathic knee pain are not only suffering from pain but also from psychiatric burden, sleeping disorders and unsatisfactory quality of life. The mean Physical SF-36 Component Summary Score in the German population aged 50–59 years was 45.27 for women and 46.70 for men. The mean SF-36 Mental Health Component Summary Score in this cohort was 50.02 for women and 51.68 for men^19^. The preoperative median PCS score of 23.05 and MCS score of 50.42 indicate considerable disease burden levels in the study population. This indicates the clinical need for an effective treatment option for subjects with neuropathic knee pain.

In recent years, PNS has gained prominence as a therapeutic option for the treatment of chronic pain^20^. This study specifically addressed the use of landmark-guided PNS implant techniques, which are less invasive than conventional neuromodulation techniques such as Spinal Cord or DRGS systems. Our data show that PNS with an HF-EMC powered implanted receiver leads to significant pain reduction in subjects with chronic knee pain. This is in line with a case-report that describes also pain improvement after PNS in a 73 year old patient suffering from chronic knee pain^21^.

This results in an improved quality of life, mood, and quality of sleep. Furthermore, opioid medication usage reduced drastically. Improvement of pain is in line with other studies that investigated the effects of PNS^8,11,18,20,22^.

The results according pain relief are comparable to DRGS and Spinal cord systems^1,8,23^. Martin et al.^11^ show a significant improvement of pain in 12 of 14 patients (85%) and a mean reduction in daily morphine dosage of 54% with a mean follow-up time of 24 months using DRG stimulation. Gilis et al*.* describe three patients suffering from chronic knee pain, that were treated with SCS. One patient showed long-term improvement of pain and decreased pain medication, the other patients required explantations of the systems^23^.

One of the main advantages of PNS over classical conservative pain medication management approaches, such as long-term therapy with opioids, is the reduced risk of side effects and medication addiction. The surgical burden is low since no systems anesthesia is necessary. Furthermore, our data show no intraoperative morbidity and/or mortality. None of the subjects reported a new post-operative sensomotoric deficit and/or an increased pain after surgery. Nevertheless, two adverse events in terms of wound infections were reported in our study.

An aspect that should be considered in future studies is the identification of subjects most likely to benefit from PNS. In our study the system was implanted without a trial: 18.2% of our subjects did not initially respond to PNS, and therefore the system was explanted. These numbers align with the study of Hunter et al.^18^ that reported 12 subjects who underwent a DRGS trial for post-TKA pain. Eight (66.6%) of these subjects showed a benefit and a permanent device was implanted. Current literature suggests that individual factors such as age, gender, and concomitant diseases may play a role. The development of predictive models and personalized therapy approaches could help maximize the efficacy of PNS while minimizing unnecessary medical interventions in subjects unlikely to benefit from this therapy^18,20^.

This study is showing improvement regarding pain and functional outcome, including quality of life as a result of treatment with a peripheral nerve stimulator system at the branches of the saphenous nerve. Nevertheless, our study is inherently limited due to its retrospective study design and the small sample size. Furthermore, while we report short-term data, long term-results are pending. Some of the study subjects reported mental discomfort due to the corona virus pandemic. This is a potential bias influencing depression and mental health scores. A further notable limitation of our study is the absence of a control group, specifically patients who underwent TKA with standard post-operative care. This absence may introduce potential biases, as comparisons with standard care or other treatment options cannot be made. Future studies should consider including control groups to better elucidate the relative effectiveness of the treatment under investigation.

## Conclusion

Externally powered peripheral nerve stimulation at the saphenous nerve branches is a minimally invasive and safe technique to treat chronic post-surgical knee pain. Our results are promising and show a considerable reduction in chronic pain, an opioid usage and improved in quality of life.

## Data Availability

The datasets generated during and/or analysed during the current study are available from the corresponding author on reasonable request.
